# Shared Oocyte Donation: Ideas and Expectations in a Bioethical Context Based on a Qualitative Survey of Brazilian Women

**DOI:** 10.1055/s-0038-1672160

**Published:** 2018-09

**Authors:** Drauzio Oppenheimer, Agatha Ramos Oppenheimer, Sthefano Teixeira de Vilhena, Augusto Castelli Von Atzingen

**Affiliations:** 1 Postgraduation program in Bioethics, Universidade do Vale do Sapucaí, Pouso Alegre, MG, Brazil

**Keywords:** oocyte donation, bioethics, infertility, reproduction, doação de oócitos, bioética, infertilidade, reprodução

## Abstract

**Objective** Assisted reproduction combines innovative technologies and new forms of procreation through gamete donation; however, it also leads to moral and ethical issues and to the wide application of referential bioethics. The objective of the present study was to understand the bioethical context of shared oocyte donation.

**Methods** The present qualitative study used the Collective Subject Discourse methodology to interview donors and recipients in Brazil.

**Results** Donors suffer from infertility, and in vitro fertilization opens the possibility of having a child; however, the cost is high, and helping the recipient is more important than the financial cost. The recipients regret delaying motherhood; adopting a child is their last option, and they desire to feel the physical stages of pregnancy. The recipients find the rules unfair regarding the lack of an oocyte bank and the fact that the treatment must be performed in shared cycles; however, oocyte donation makes it possible to realize the common dream of motherhood.

**Conclusion** The obtained data showed that the patients are suffering and frustrated due to infertility, and they realize that in vitro fertilization may be the treatment they need. These women believe that children are essential in the constitution of the family, and scientific advances bring about innovative technologies and new forms of family constitution, with repercussions in the social, economic, political, and family contexts that lead to bioethical questions in Postmodernity.

## Abstract (pt)

**Resumo:**

**Objetivo** A reprodução assistida agrega tecnologias inovadoras e novas formas de procriação por meio da doação de gametas; no entanto, também leva a questões éticas e morais e à ampla aplicação da bioética referencial. O objetivo deste estudo foi compreender o contexto bioético da doação compartilhada de oócitos.

**Métodos** Este estudo qualitativo utilizou a metodologia do Discurso do Sujeito Coletivo para entrevistar doadoras e receptoras no Brasil.

**Resultados** As doadoras sofrem de infertilidade, e a fertilização in vitro abre a possibilidade de ter um filho; no entanto, o custo é alto, e ajudar a receptora é mais importante do que o custo financeiro. As receptoras se arrependem de retardar a maternidade; adotar uma criança é sua última opção, e elas desejam sentir os estágios físicos da gravidez. As receptoras consideram as regras injustas em relação à falta de um banco de oócitos e ao fato de que o tratamento deve ocorrer em ciclos compartilhados; no entanto, a doação de oócitos possibilita a realização do sonho comum da maternidade.

**Conclusão** Os dados obtidos mostraram que as pacientes estão sofrendo e frustradas devido à infertilidade, e percebem que a fertilização in vitro pode ser o tratamento de que necessitam. Essas mulheres acreditam que as crianças são essenciais na constituição da família, e os avanços científicos agregam tecnologias inovadoras e novas formas de constituição familiar com repercussões nos contextos sociais, econômicos, políticos e familiares que levam a questões bioéticas na Pós-modernidade.

## Introduction

Fecundation and the desire to have children and to form a family have always had a divine connotation. In the 1970s, rumors surfaced that scientists could play God by performing ‘conception’ in the laboratory rather than by sexual intercourse, causing concern to religious groups and to those who prioritize ethical behavior in society. This moment, which coincides with the emergence of bioethics, demonstrates the type of concern that guides the reflection in this field, which has grounds for action in the area of reproductive health.^1^

Bioethics is a new discipline that establishes a bridge between the scientific and humanistic cultural dimensions^2^ and combines human values and biological knowledge. It is a new ethical science that combines humility, responsibility, interdisciplinarity, and intercultural competence, thus enhancing humanity; it is based on the study of human conduct in the life sciences and health care considering moral principles and values.^3^ The technological advances in reproductive medicine, particularly in vitro fertilization (IVF), with the manipulation, production, and freezing of embryos and experimentation with embryos, refers to the ethical, legal, and ontological statute of human life since its inception and development.^4^ Therefore, bioethics is linked to assisted reproduction, embryo genetic studies, cloning, embryonic stem cell research, organ donation, human participation in experimentation, gene therapy, gene sequencing, and end-of-life issues.^5^

These questions are based on the principles of bioethics related to principlism.^6^ Principlism is not a theory or an ethical doctrine; rather, it defines the ethical basis for the protection of human beings by mediating tools in the deliberation and decision of bioethical subjects and questions, helping with self-regulation and legislation.^7^ It is based on four main pillars of autonomy, nonmaleficence, beneficence, and justice.

Autonomy is the principle of greater weight in bioethics; it reflects respect for a person's will according to their point of view, values, and convictions. In the medical practice, professionals should respect their patient's autonomy to consent to diagnostic testing, procedures, and treatments. Nonmaleficence is a fundamental Hippocratic principle of medical ethics, *primum non nocere* – first, do no harm. It is a moral requirement of the medical profession not to incur malpractice or negligence; the expected benefit should outweigh the known risks. In turn, beneficence is the moral obligation to benefit others whether it is desired or not, and is within the Hippocratic oath. And finally, justice refers to the possibility of accessing services and resources in an egalitarian way to equate the opportunities made available to all human beings in the same conditions. Dignity, freedom, equity, solidarity, and dialogue are other topics of principlism that must be considered.^8^

Therefore, we see an intimate relationship between bioethics and oocyte donation, which is a growing practice in assisted reproductive treatments. Donors and recipients deserve a detailed reflection because of this approach. Traditionally, the birth of a child symbolizes a ‘divine gift,’ and assisted reproduction (AR) counteracts this concept. Numerous philosophical questions and doubts arise with the technological advances related to the AR techniques (ARTs) that are available for infertile couples.

Infertility, which is defined by the World Health Organization (WHO) as failure to achieve pregnancy after one year of unprotected sex, is a public health problem. However, infertility raises issues regarding individual and family well-being and the social insertion of couples,^9^ and ARTs are an alternative method to achieve procreation.^4^ From the list of problems associated with infertility, ovulatory factors comprise the main female cause.^10^ Today, because of work overload, eating habits, and lifestyle choices, a significant percentage of women are beginning menopause at an early age. Moreover, with maternity occurring later in life, there is an increased incidence in infertility due to ovarian failure, and ARTs enable procreation in this context.^11^ Furthermore, IVF using donated oocytes becomes a tool to assist with the reproductive goal in cases of ovarian failure.^9^

Despite several advances in recent years, reproductive medicine and ARTs are not regulated by specific laws in Brazil. Rather, they are regulated by ethical resolutions of the Brazilian Federal Medical Council (Conselho Federal de Medicina, CFM, in the Portuguese acronym), which have the same effect as laws for the medical class. Spermatozoa were the first germ cells successfully used in donation procedures, and Brazil currently has sperm banks, with costs for those who use donor semen.^12^

Oocyte donation is more complex; it involves higher costs and presents risks to the donor due to the need for ovarian stimulation and surgical oocyte collection. In Brazil, embryo and gamete donations are regulated by the CFM. The Ethical Directions for Assisted Reproductive Techniques (CFM Resolution no. 1358/1992)^13^ has allowed gamete donation since 1992, but it has not established clear rules for its practice. Almost 20 years after its first publication, in 2010, the CFM published (CFM Resolution no. 1957/2010)^14^ an update that included no modifications about gamete donation. Only in the update published in 2013 (CFM Resolution no. 2013/13)^15^ did the CFM clarify this issue, establishing that voluntary gamete donation was allowed in the form of shared oocyte donation, in which both donor and recipient undergoing infertility can share oocytes and the financial costs of the treatment.^15^ However, in 2015, a new resolution established that voluntary donation was permitted only for male gametes (spermatozoids), and that oocyte donation was only allowed in a shared model, in which donors and recipients participate in IVF.^16^ The latest resolution, which was published in 2017 (CFM Resolution no. 2168/2017),^17^ stablished the permission to voluntarily donate semen and oocytes, as well as the shared oocyte donation, which was similar to the 2013 resolution.

Due to the complexity of voluntary oocyte donation and to the modifications in the CFM resolutions, oocyte banks are difficult to establish. Shared donation in IVF cycles, in which donors and recipients participating in ARTs share biological material and financial costs, is common, and has been allowed since 2013. Because there are many more recipients than donors, the recipients must wait to receive oocytes in AR clinics. Accordingly, conflicting and innovative situations arise regarding the rights and duties of those who seek AR clinics for procedures performed with donated gametes, giving rise to bioethical questions that merit analysis. The bioethical view is indispensable to examine situations, postures, and procedures when considering how to avoid injustice and inequality.^4^

A recent systematic review about the motivations and experiences of oocyte donors, recipients, and egg sharers demonstrated that the attitudes and feelings of patients involved on all sides of the donation process were extremely positive. Moreover, the motivations for the donors were the wish to help another couple have a child and to obtain a cheaper treatment, which are equally important.^18^

The present study evaluated the ideas and expectations of oocyte donors and recipients in an egg-sharing program in Brazil. The data was collected through a semi-structured questionnaire, and the researchers discussed the viewpoints of the studied population in a bioethical argument regarding AR issues that involve medical professionals, patients, society, and the entities responsible for the resolutions.

## Methods

The present study was developed as a collaboration between Universidade do Vale do Sapucaí and Pró Criar, a private reproductive medicine clinic, both located in the city of Pouso Alegre, state of Minas Gerais, Brazil. This study received approval from the Ethics Committee of the university (Univás, protocol no. 61451416.8.0000.5102), and all patients signed a free and informed consent form, guaranteeing their anonymity.

A total of 20 candidates for a shared oocyte donation program (10 oocyte recipients, 10 oocyte donors) who were examined at Pró Criar were consecutively invited to participate and answer the questionnaire between November 2016 and January 2017. All 20 invited women agreed to participate and answer the questions, and were included in the study. No patients were excluded.

The present qualitative study employed the Collective Subject Discourse (CSD) methodology for data analysis and systematization.^19^ A semi-structured questionnaire asked the following two questions: ‘What does it mean to you to be an oocyte donor/recipient?’; and ‘What are your expectations regarding infertility and oocyte donation?’

These questions were previously validated and applied to two patients (one oocyte donor, one oocyte recipient) to verify whether they actually provided the data that the researchers meant to collect.^20^ After validation, the interviews were performed with the 20 patients (10 donors, 10 recipients). The interviews lasted an average of 30 minutes. The patients had no connection to one another, and the interviews were conducted individually, recorded, and later transcribed for analysis.

## Results

The participants' demographic characteristics are described in Table 1. According to Brazilian law, the oocyte donor must be ≤ 35 years of age, and most oocyte recipients are of advanced maternal age. Thus, as expected, the women in the donor group were younger than the recipients, and, consequently had infertility for a shorter period of time. All women were married, and most had college degrees (70% of each group). The most common cause of infertility among the oocyte recipients was ovarian failure; as or the oocyte donors, in most cases, the infertility did not affect them, but their partners.

**Table 1:** Mean demographic characteristics of the study participants

	Donors	Recipients	*p*-value
Age (years)	29.7 ± 2.5	41.1 ± 4.9	< 0.001
Body mass index (kg/m^2^)	23.4 ± 2.1	24.9 ± 2.3	0.145
Infertility time (years)	2.7 ± 0.7	5.0 ± 2.6	0.014

The results of this study were obtained through key expressions (KEs), central ideas (CIs), analysis, and a discussion about the interview contents. From the patients' transcribed responses, the KEs of each discourse were categorized into CIs and quantified as percentages of the total number of patients. Table 2 describes the CIs regarding question 1 (‘What does it mean to you to be an oocyte donor/recipient?’), while Table 3 describes the responses for question 2 (‘What are your expectations regarding infertility and oocyte donation?’).

**Table 2:** Central idea categories for Question 1: What does it mean to you to be an oocyte donor/recipient?

Central idea	Donors, n (%)	Recipients, n (%)	*p*-value
Acceptance			
Accepted the donation/reception	4/10 (40%)	6/10 (60%)	0.656
**View with caution**			
Difficulty accepting donation/reception	4/10 (40%)	3/10 (30%)	1.000
Lower treatment costs	3/10 (30%)	0/10 (0%)	0.211
Considered donation the same as donating a son/daughter	2/10 (20%)	3/10 (30%)	1.000
Concerned about donor characteristics	0/10 (0%)	1/10 (10%)	1.000
Afraid of treatment success	0/10 (0%)	3/10 (30%)	0.211
**Motivation**			
Possibility of motherhood	7/10 (70%)	10/10 (100%)	0.211
Donation due to lower treatment costs	7/10 (70%)	2/10 (20%)	0.070
Donation/reception to help other women	8/10 (80%)	6/10 (60%)	0.629
Imagined another woman's position	6/10 (60%)	2/10 (20%)	0.170
Male infertility	2/10 (20%)	0/10 (0%)	0.474
Oocyte surplus	2/10 (20%)	1/10 (10%)	1.000
Desiring physical/emotional motherhood stages	1/10 (10%)	6/10 (60%)	0.057

**Table 3:** Central idea categories for Question 2: What are the expectations regarding infertility and oocyte donation?

Central ideas	Donors, n (%)	Recipients, n (%)	*p*-value
Expectations			
Search for alternatives to infertility	4/10 (40%)	3/10 (30%)	1.000
Donation due to lower treatment costs	4/10 (40%)	2/10 (20%)	0.629
In vitro fertilization to achieve motherhood	9/10 (90%)	8/10 (80%)	1.000
Donation of oocytes as a last option	1/10 (10%)	5/10 (50%)	0.033
Fear regarding treatment success	2/10 (20%)	2/10 (20%)	1.000
Oocyte donation as an exchange of favors	3/10 (30%)	0/10 (0%)	0.211
Oocyte donation to help other women	8/10 (80%)	4/10 (40%)	0.170

### Question 1: What Does it Mean to You to be an Oocyte Donor/Recipient?

Three CI subgroups were created based on the participants' responses regarding the meaning of being a donor/recipient (question 1): 1) the positive meaning (acceptance) subgroup included the discourses that did not express any restrictions; 2) the view with caution subgroup included discourses in which some restrictions or difficulties regarding acceptance were expressed; and 3) the motivation subgroup included the CIs that lead to the reasons why the women opted to be oocyte donors/recipients (Table 2).

The acceptance of oocyte donation was mentioned by 40% of the donors and by 60% of the recipients, which shows that, on average, half of the women did not present difficulty accepting egg sharing. Those who had some initial restrictions expressed difficulty accepting the donation, fear that the lower treatment costs due to egg sharing are not significant for donors, and concern about the treatment success of the recipients. Approximately 25% of the women (20% of donors and 30% of recipients) initially interpreted oocyte donation as the donation of a son/daughter.

Regarding the motivations for treatment, the two groups demonstrated that the possibility of motherhood was the primary reason for oocyte donation/reception, as both donors and recipients were infertile women seeking ARTs to become pregnant and realize their dream of being mothers. This motivation was reinforced by the statement that most donors (80%) agreed to donate to help another woman, and 60% of the recipients stated that they desired to feel the physical and emotional stages of motherhood. The financial issue was also important, primarily for the donors (70%), because they share the costs of the treatment with the recipients.

### Question 2: What are your Expectations regarding Infertility and Oocyte Donation?

Regarding the expectations about infertility and oocyte donation (question 2), the KEs were categorized into CIs and quantified as percentages of patients. The following subgroup was created: 1) expectations related to oocyte donation/reception (Table 3).

The CI concerning the expectations was the assertion that IVF would be the means to realize the dream/desire of motherhood (70% of donors, 100% of recipients). A total of 60% of the patients stated aiding another woman as the expectation, and this occurred more frequently among the donors (80%). A total of 30% of the donors mentioned the fact that oocyte donation works as an exchange of favors.

## Relevant Issues Mentioned during the Interviews

Among the relevant issues raised in the patients' discourse for questions 1 and 2, the most preponderant discourse included the anguish, sadness, and frustration generated by infertility, which demonstrates the importance of infertility among the emotional issues of women. In this context, reports that ARTs bring emotional relief stand out. The women also reported the lack of adequate information about ARTs and the high cost of the treatment. The issue of adoption only after all ART resources were exhausted was mentioned by 40% of the patients (both donors and recipients). An average of 50% of the women questioned the rules and difficulties for oocyte donation/reception in Brazil, and did not find them fair.

## Discussion

Gamete donation consists of the use of gametes donated by couples who cannot procreate but wish to have children.^21^ Spermatozoa were the first germ cells used in ARTs with donated gametes. In sperm banks, donors voluntarily cooperate altruistically after various laboratory examinations, and the semen is marketed by authorized clinics.^22^ The Brazilian rules for oocyte donation were not clear during the first years of its practice in the country, and many changes were made in the past few years.^14^ ^15^ ^16^ ^17^ In the period in which this study was performed (2016–2017), only the egg-sharing model was allowed in IVF cycles, in which donors and recipients with reproductive problems shared both biological material and financial costs.^16^ According to the CFM, the donor age limit is 35 years; anonymity is mandatory; and one should seek greater phenotypic similarity and compatibility between donors and recipients, and not produce 2 pregnancies of different sexes in an area of 1 million inhabitants with donor oocytes. The Resolution of the CFM also advocates 50 years of age as a limit for IVF recipients.^16^ ^17^

Scientific advances, economic globalization, international dialogue, and cultural factors have created ethical and moral questions that impose limits on procedures performed in human beings. Bioethics arises out of this multicultural set of factors,^23^ and it is based on the study of human conduct in the life sciences and health care, considering moral principles and values.^3^ Bioethics includes safeguarding individual rights and protecting human beings from the deleterious effects of technology, while simultaneously ensuring their access to fundamental advances.^24^ The technological advancements in reproductive medicine, particularly in IVF with embryo manipulation, are related to the ethical, legal, and ontological statute of human life since its inception and development.^4^ Accordingly, we see an intimate correlation between bioethics and oocyte donation, the central theme of the present study.

The results of the present research reveal the ideas of donors and recipients regarding shared oocyte donation. The KEs of the transcribed speeches were categorized into CIs and quantified and grouped into social representations of acceptance, issues with caution, motivation, and expectations. We also highlighted the relevant issues that were raised in the responses of the interviewed women, and the suffering caused by infertility was the most prominent point. The lack of adequate knowledge of AR resources, such as oocyte donation/reception and shared cycles, reveals the need for multidisciplinary support and to provide clear information to couples, respecting their autonomy and their freedom to decide how they will seek treatment for their infertility. A systematic review of European studies also identified a lack of knowledge of egg-sharing outside of fertility clinics, which was expressed by a minority of patients, and concerns about whether participating in the egg-sharing program would impact their success were raised,^25^ as they were in the present study.

The CIs of helping other women and financial issues are some of the points highlighted by the donors, while the main motivation expressed by the recipients was their desire to feel the physical and emotional stages of motherhood. The desire to help others and the financial advantages are common motivations also described by other authors studying egg-sharing patients.^26^ However, our data demonstrated that the maternal desire was the leading factor for both donors and recipients, as is expected among women undergoing ARTs to have a child. This desire for a family should be valued without compromising autonomy and freedom.^27^

Donors, even those suffering from infertility, perceive that the recipients' situation is worse, and, out of solidarity, dignity, beneficence, justice, dialogue and equity, are willing to donate their oocytes. However, the financial contribution of the recipients to the donors, which enables their treatment, must also be considered.^28^ The high cost often makes treatment impossible, leaving the patients that cannot afford the treatment frustrated, which raises the issue of social inequality,^29^ an important issue in countries like Brazil, where there are very restricted numbers of public ART programs, and the costs of treatment in a private clinic are high. The recipients feel the physical need to become pregnant and raise a child; these women compare oocyte donation with organ donation, and mention the lack of other options. Other studies cited in a systematic review of European populations reported that women would prefer to use their own oocytes in the first place, but this was not feasible.^18^

The CIs for the question regarding the expectations reveal the realization of the common dream of motherhood as the most common response among donors and recipients. For the recipients, the delay in motherhood forces them to seek ART clinics, where they will have the possibility of fulfilling the dream of pregnancy by receiving oocytes. However, they feel hurt by the obligation to perform the procedure in shared cycles; these women were unaware of the rules and did not know that oocyte banks are not available; they had imagined equal and fair rules. Moreover, adoption is not their first choice because they want to feel the physical stages of pregnancy.

Our data also demonstrated that most of the same motivations, concerns, and expectations were mentioned by both donors and recipients, which demonstrates that both groups have similar views regarding egg-sharing programs. Gürtin et al^30^ showed that donor and recipient experiences and opinions have very few differences, and highlighted the positive assessment of the egg-sharing program, and the words that best described the experience were *rewarding* and *satisfying*. However, this study evaluated patients who underwent treatment, and our study interviewed women before treatment. Thus, the experiences about egg-sharing described by Gürtin et al^30^ differ from the ones we noted, as we observed both positive motivations and concerns.

Objective criteria must be determined to reduce inequalities. The limited number of donors and the financial costs of the treatment should be evaluated by the authorities and professionals involved in seeking justice and breaking down barriers.^29^ The current ARTs, such as shared oocyte donation, are a wide bioethics field of discussion that involves both donors and recipients.^31^ An ethical discussion arises regarding the fact that donor care can be can be ethically compromised by the conflict of interests and incentives inherent in the current egg-sharing program in the IVF process, as not all donors undergo an adequate informed consent process.^32^

Donors and recipients deserve detailed reflection because of this approach. These questions are based on the principles of bioethics related to principlism.^6^ The reference to principlist bioethics is important to the professionals working in AR and the patients (donors and recipients) because it enables a deep reflection of ART methods. In 1979, due to multidisciplinary dilemmas and bioethical problems, Tom Beauchamp and James Childress published the book *Principles of Bioethical Ethics*.^33^ In it, they proposed a theory based on four basic principles of beneficence, autonomy, justice, and nonmaleficence, which became the foundations of the bioethics principles that are widely used in clinical bioethics in Brazil. By urging compliance, this set of ethical norms was called principlism by Dan Clouser and Bernard Gert in 1990.^34^ These principles complement one another and are rules for guiding actions against the bioethical dilemmas of present-day society that occur as a result of the advancements in biological and medical sciences.^35^

The references of bioethics should be included in the discussion to formulate resolutions and laws in the face of advances in technology and science, seeking egalitarian rules for beneficence, autonomy, freedom, solidarity, equity, nonmaleficence, dialogue, and justice.^12^ Advancements have been made in the CFM rules regarding oocyte donation: voluntary donation that is not necessarily shared was first authorized in 2017. This resolution makes egg donation similar to sperm donation regarding the freedom of a fertile woman to choose to donate, and it enables the creation of an oocyte bank.

## Conclusion

The objective of the present study was to understand the motivations and expectations of patients regarding shared oocyte donation and to foster a discussion about this topic from a bioethical perspective. The data obtained showed that infertile women are generally frustrated due to the inability to become pregnant; they search for information, and believe the IVF may be the treatment they need. They want to have a child, and are not against adoption, but desire to feel the physical and emotional stages of pregnancy. They face the high cost of the treatment, and oocyte donation/reception appears as a way to reduce the costs for the donor and to enable treatment and the recipients to become pregnant. They note that it is necessary to share the treatment, which makes it difficult and time-consuming; they also stated that the rules are not fair and egalitarian, and that financial assistance is not substantial and becomes a secondary condition. Solidarity, with mutual help, is the most important factor in the quest to achieve the common dream of being a mother. These women believe that children are essential in the constitution of the family, and scientific advances and innovative technologies enable the constitution of new forms of family, which has repercussions in the social, economic, political, and family contexts, leading to modern bioethical questions. New medical technologies have created uncertainty and new questions regarding moral and ethical principles; thus, this creates the possibility to practice a bioethics that aims to join scientific knowledge and human values within a multidisciplinary approach, particularly in ARTs. Bioethics references should guide and assist in the formulation of resolutions in assisted human reproduction. Further studies are needed to add knowledge and encourage debate regarding this current and comprehensive issue.
